# Serum LAIR-2 Is Increased in Autoimmune Thyroid Diseases

**DOI:** 10.1371/journal.pone.0063282

**Published:** 2013-05-14

**Authors:** Rita Simone, Giampaola Pesce, Princey Antola, Domenico F. Merlo, Marcello Bagnasco, Daniele Saverino

**Affiliations:** 1 Department of Experimental Medicine – Section of Human Anatomy, University of Genova, Genova, Italy; 2 Departments of Medicine and Cell Biology, North Shore University Hospital, Manhasset, New York, United States of America; 3 Autoimmunity Unit, Department of Internal Medicine, University of Genova, Genova, Italy; 4 Epidemiology, Biostatistics and Clinical Trials, IRCCS AOU San Martino, IST – Istituto Nazionale per le Ricerca sul Cancro, Genova, Italy; University of Michigan Medical School, United States of America

## Abstract

Leukocyte-associated Ig-like receptor (LAIR) is a small family-receptor able to inhibit immune cell function via collagen binding. It exists as both membrane-bound and soluble forms. LAIR-1 functions as an inhibitory receptor on natural killer cells, T lymphocytes and monocytes. In addition to LAIR-1, the human genome encodes LAIR-2, a soluble homolog. Several studies have focused on LAIR-1, whereas few investigations concentrate on the expression and function of LAIR-2. We demonstrate the presence of high LAIR-2 levels in 74/80 sera from patients with autoimmune thyroid diseases (both Graves’ disease and autoimmune thyroiditis). LAIR-2 levels seemed not to be related to specific clinical manifestations, such as thyroid functions (hypo- or hyperthyroidism), or specific clinical features (such as ophtalmopathy). In addition, serum LAIR-2 is able, *in vitro,* to bind its natural ligand, collagen. Since LAIR-2 has been found to have higher affinity for collagens than LAIR-1 did, we hypothesize a potential regulating capability of serum LAIR-2 in finally regulating the inhibitory capability of LAIR-1.

## Introduction

The regulation of immune responses is the outcome of a balance between positive signals that trigger them and inhibitory mechanisms that prevent excessive clonal expansion and autoimmunity. Therefore, a prevalence of activation should render T cells responsive to antigens, whereas a prevalence of inhibition should lead to T-cell anergy. T-cell activation requires a primary signal delivered by the antigenic peptide presented by major histocompatibility complex (MHC), and a non-specific signal that can finely regulate the level of activation or inhibition [1]. Inhibitory signals are required to terminate an immune response and to prevent excessive immune reactions or autoimmune disease [1]. These signals can be provided by inhibitory immunoreceptors, often containing immunoreceptor tyrosine-based inhibitory motifs (ITIMs) in their cytoplasmic tails [1]. Immune cells express several inhibitory receptors, differentially expressed on immune cells: their expression is known to change upon cellular activation [2]. Another regulation pathway is provided by secretion of soluble receptor variants retaining their ligand binding capacity. Soluble receptors can be obtained either by proteolytic cleavage/shedding or alternative splicing of mRNA transcripts [3]. For example, soluble CTLA-4 shows immunoregulatory functions via binding to CD80/CD86 molecules and acts indirectly either as inhibitor or as enhancer of immune response [4].

The leukocyte-associated Ig-like receptor is a small family of ITIM-containing inhibitory receptor, belonging to the Ig superfamily [5]. LAIR-2, is a secreted molecule, whereas LAIR-1 is its membrane bound homologue, LAIR-2 is believed to play a regulatory role in the interaction between collagen and LAIR-1 [6], [7]. LAIR-2 is encoded by a gene located near the LAIR-1 gene in the leukocyte receptor complex (LRC) on human chromosome 19q13.4 [5]–[9], suggesting that these molecules have evolved from a common ancestral gene. LAIR-2 protein has a single Ig-like domain sharing 84% sequence homology with LAIR-1, and lacks transmembrane and cytoplasmic regions suggesting that it is a secreted protein [5], [7]. LAIR-1 is a type I transmembrane glycoprotein of 287 amino acids containing a single extracellular C2-type Ig-like domain and two ITIMs in its cytoplasmic tail. LAIR-1 is expressed on the majority of immunocytes [5], [8], [10]. Cross-linking of LAIR-1 in vitro delivers a potent inhibitory signal [5], [8], [10]. Recently, collagens were identified as natural, high-affinity ligands for LAIR molecules [9]. Interaction of LAIR-1 with collagens directly inhibits immunocyte activation in vitro and may represent a key mechanism of peripheral immune regulation through extracellular matrix [9]. Given the broad expression profile of LAIR-1 [5] and the abundance of collagen in the human body, a fine-tuned regulation of collagen-LAIR-1 interaction is likely needed. As above mentioned, the soluble LAIR-2 molecule present in the plasma and interstitial fluid is likely to play a significant role in such regulation.

Importantly, a role for LAIR-2 in systemic autoimmunity is suggested by the observation of increased LAIR-2 levels in synovial fluid of patients with rheumatoid arthritis [6], [11] and ankylosing spondylitis [12]. However, data concerning the possible role of LAIR-2 and LAIR molecules/collagen interactions in organ-specific autoimmunity are lacking. Autoimmune thyroid diseases (ATD), namely, autoimmune (Hashimoto’s) thyroiditis and Graves’ disease, are the most frequent organ-specific autoimmune diseases, and autoimmune thyroiditis is regarded as a prototype of such pathological conditions. Here we report the presence of high levels of LAIR-2 in sera of patients with autoimmune thyroid diseases.

## Materials and Methods

### 2.1 Serum Samples

Serum samples were obtained from 80 patients with autoimmune thyroid diseases (ATD) (28 males, 52 females, age range 22–60). Written informed consent was obtained from all the patients; all samples were obtained following the ethical guidelines of the most recent Declaration of Helsinki (Edinburg 2000) and approved by the Ethics Committee of Ospedale San Martino, Genova, Italy. Twenty-one of them had Hashimoto’s thyroiditis (HT), 59 Graves’ disease (GD). The very large majority of ATD patients had detectable levels of auto-antibodies to thyroid peroxidase (77 out of 80), 51 out of 80 had also auto-antibodies to thyroglobulin. The diagnosis was formulated on the basis of commonly accepted clinical and laboratory criteria, including thyroid ultrasonography, serum TSH and free thyroid hormone levels, anti-thyroid peroxidase antibodies, and anti-TSH receptor antibodies and thyroid scintigraphy (for GD). Patients with HT were either mild hypothyroid (raise TSH with low-normal free thyroxine) or euthyroid under L-thyroxine replacement. Patients with GD were hyperthyroid (from mild to severe) and were studied before starting antithyroid therapy: 41 of them had clinically apparent Graves’ ophthalmopathy. Finally, from a group of 20 GD patients serum samples were collected also 1 month after radioiodine therapy. In these patients euthyroidism or post-treatment hypothyroidism was documented, as well as thyroid gland volume reduction (by ultrasonography).

As control, we collected sera from 10 patients with non-autoimmune hyperthyroidism (toxic adenoma or multinodular toxic goiter, MNTG, which are closely related non-autoimmune thyroid diseases with hyperthyroidism caused by autonomously functioning thyroid nodules) and from 25 healthy donors.

Free thyroid hormone concentrations were assayed by routine ELISA methods, as well as TSH (3^rd^ generation) and Thyroid Peroxidase and Thyroglobulin autoantibodies.

### 2.2 ELISA

**Figure 1:**
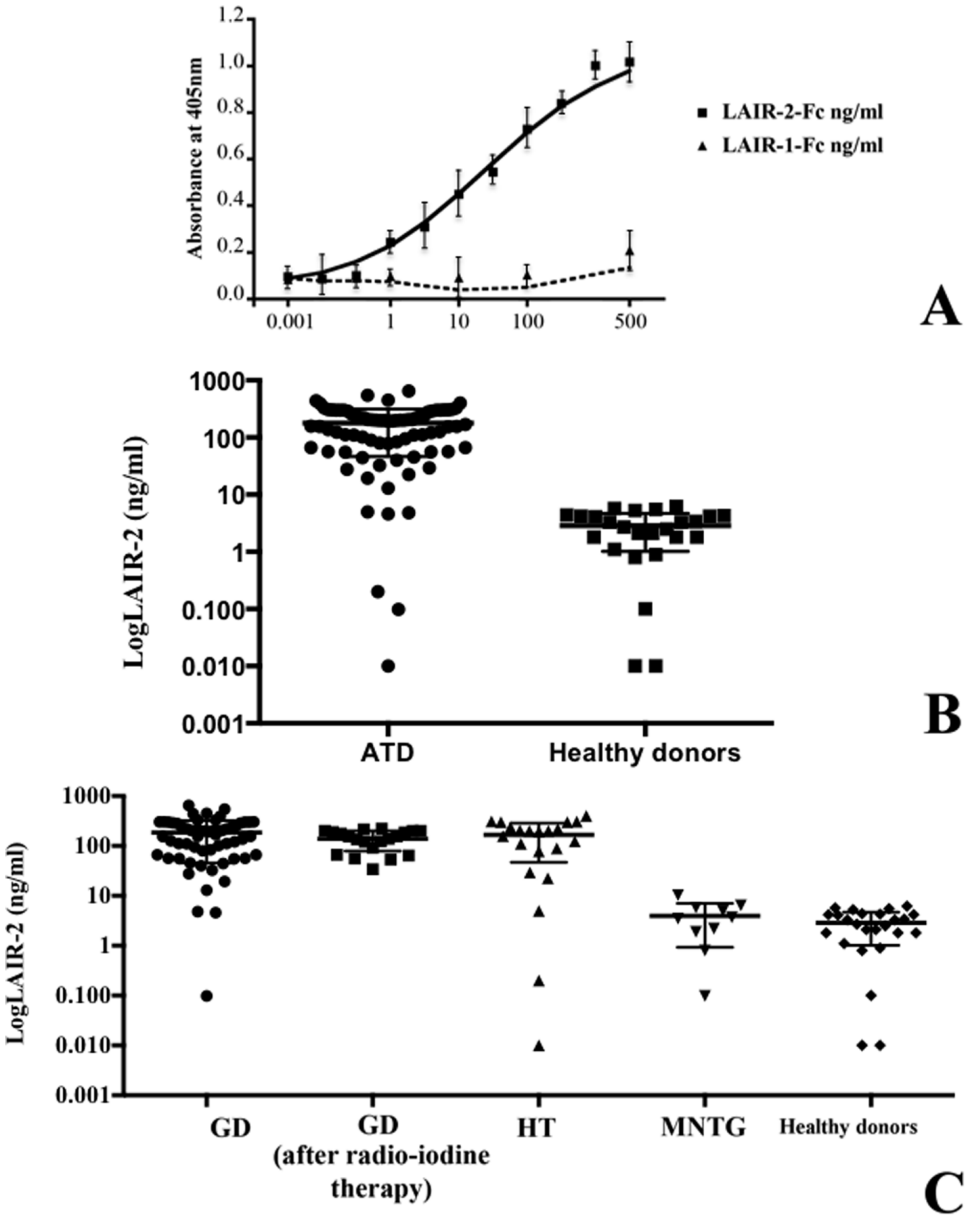
LAIR-2 is found in serum of patients with ATD and healthy donors but its level correlates with autoimmune disease. *A,* A calibration curve of LAIR-2 ELISA was performed. The plotted OD values were obtained with serial dilution of LAIR-2-Fc protein. In addition, no relevant cross-reaction with soluble LAIR-1-Fc was detectable. A representative experiment of three is shown. Bars represent standard deviation. *B,* Serum LAIR-2 is measurable both in healthy donors and patient with ATD, otherwise ATD had far higher circulating LAIR-2 levels. *C,* LAIR-2 ELISA on 59 sera of GD, 20 of GD patients one month after radio-iodine treatment, 21 HT patients, 10 of patients with MNTG and 25 healthy donors. Samples were diluted 1∶10 and tested in triplicate (deviation between triplicates <10%; detection limit 0.1 ng/ml).

Serum LAIR-2 was measured by ELISA. Ninety-six-well MAXIsorb flat-bottom plates (Nunc) were coated overnight at 4°C with anti-LAIR-2 mAb (IgG2b, clone 7H82, Sichim) (5 µicrog/ml in 50 µicrol/well PBS). After washings, plates were incubated with PBS-3% BSA to block aspecific interactions. After three washes, sera were assayed for presence of the LAIR-2 protein. Human recombinant LAIR-2-Fc construct (Lair-2 fused to the Fc region of human IgG, GeneBank ID: NP002279, R&D Systems) serially diluted from 200 ng/ml was used as reference. Samples were diluted in PBS-3% BSA and incubated 2 h at room temperature. After three washes, the wells were incubated with biotinylated LAIR-2 mAb (IgG2b, clone 319701, R&D Systems) 2 h at room temperature. After washings, wells were incubated with StreptABComplex/HRP (DakoCytomation) 1 h and colour development was performed by adding 100 µicrol/well ABTS (Roche Diagnostics). Specificity of the assay and exclusion of cross-reaction with soluble LAIR-1 [13] was stated by the producer and confirmed in our laboratory by dedicated experiments, were LAIR-2 mAb was tested on coated plates with LAIR-2 Fc or with LAIR-1 Fc (Lair-1 fused to the Fc region of human IgG, GeneBank ID: 3903, R&D Systems) separately (Fig. 1A, see also below).

Each sample was diluted 1∶10 and tested in triplicate. Deviation between triplicates was <10% for any reported value.

### 2.3 Collagen Binding Test

Furthermore, we designed experiments to demonstrate that sera from ATD patients are able to effectively bind collagen via LAIR-2 [6], [7]. The experimental design takes into account that the mAb used is directed against an epitope of LAIR-2 distinct from, and independent of the collagen-binding site. MAXIsorb flat-bottom plastic plates were coated overnight with sera from a selected group of patients and controls. After washing and blocking of non-specific plastic binding with PBS-3% BSA, Type 1 collagen (Sigma, 2 µicrog/ml) was added and further incubation carried out (3 h at 4°C). Thereafter, collagen solution was carefully removed and transferred into uncoated wells, incubated as above, then the wells were washed and incubated with saturating BSA solution as above. In order to quantify the collagen recovered, a further incubation (3 h at 4°C) with the human recombinant LAIR-2-Fc construct (20 µicrog/ml) was carried out, followed by washing and addition of biotin-labelled anti-human IgG (Fc) antiserum (eBiosciences) followed by StreptABComplex/HRP for 1 h, then substrate was added. Control wells were set up with non pre-incubated collagen solution.

At variance, LAIR-2-Fc construct was added on plastic plates coated with sera, and incubated (3 h at 4°C). Then, supernatants were carefully removed and transferred into uncoated wells and incubated as above. In order to quantify the LAIR-2-Fc recovered, a further incubation with Type 1 collagen followed by washing and addition of biotin-labelled anti-Type 1 collagen mAb (clone I-8H5) followed by StreptABComplex/HRP for 1 h, then substrate was added.

### 2.4 Statistical Analysis

Log transformed LAIR-2 levels were used to test differences between groups of patients and in healthy donors. The Welch test [14] was used to account for heterogeneity of variances in study groups. Post-hoc analyses was carried out accounting for multiple comparisons (Bonferroni correction). Differences were considered statistically significant at a p value <0.05. Statistical analysis was performer by using IBM-SPSS Statistical software, version 20.

## Results

### 3.1 Plasma LAIR-2 Levels in Patients with ATD

In order to verify the possible role of LAIR-2 in the pathogenesis of autoimmune diseases, we investigated the presence of LAIR-2 in a group of ATD patients and controls.

LAIR-2 was detectable in the very large majority of healthy volunteers. Twenty-three out of 25 sera from healthy controls (92%) had detectable LAIR-2 (0.1–6.2 ng/ml). This is at variance with previous observation [6] possibly due to different mAb used. In addition, 74 out 80 ATD patients had far higher circulating LAIR-2 levels (4.6–450 ng/ml) (p<0.05). By contrast, a similar percentage of positive sera were observed (94%) (Fig. 1B). There was no difference between patients with HT and GD (Fig. 1C). In addition, LAIR-2 concentrations were significantly higher in patients with hyperthyroid GD than in controls with non-autoimmune hyperthyroidism (p<0.05) (Fig. 1C, MNTG). There was no relationship between sex and age and LAIR-2. In fact, LAIR-2 mean level was similar in males (200.2 ng/ml±121.7) and females (206.9 ng/ml±141.0) (p = 0.873) and was not associated with age (Pearson correlation coefficient = −0.03; p = 0.85). We found no relationship in GD between LAIR-2 and hyperthyroidism severity, or presence/severity of ophthalmopathy, or thyroid auto-antibodies (as also in HT, see Table 1). Of interest, the analysis of sera collected from patients at different time points (before and 1 months after radio-iodine therapy) showed that the ability to produce LAIR-2 is not related to thyroid functions. As shown if Fig. 1C, no statistical differences in serum LAIR-2 levels among GD patients before and after radio-iodine therapy were evident. In addition, post hoc analysis confirm these results showing significantly increased LAIR-2 mean levels in GD and HT and in GD patients after radio iodine therapy than in healthy donors, in GD and HT than in MNTG patients, and in GD patients after radio iodine therapy than in MNTG patients. Thus, it can be envisaged that the capability to produce LAIR-2 is genetically determined, and not merely dependent on the clinical manifestations, or to disease evolution.

**Table 1:** No relationship between LAIR-2 and thyroid auto-antibodies was evident.

	LAIR-2 (ng/ml)	TGA (U/ml)	TPO (U/ml)
	range (median)	range (median)	range (median)
GD pre	0.1–450 (110,3)	14->5000 (610)	6->3000 (635,8)
		Spearman r = 0.26	Spearman r = 0.17
		p = 0.17	p = 0.47
GD post	4.6–400 (93.7)	9–4912 (683.8)	17->3000 (548.6)
		Spearman r = 0.22	Spearman r = 0.07
		p = 0.47	p = 0.75
HD	0.01–401.5 (193.1)	21–5000 (915)	12.1->3000 (527.4)
		Spearman r = 0.23	Spearman r = 0.15
		p = 0.48	p = 0.68

**Figure 2:**
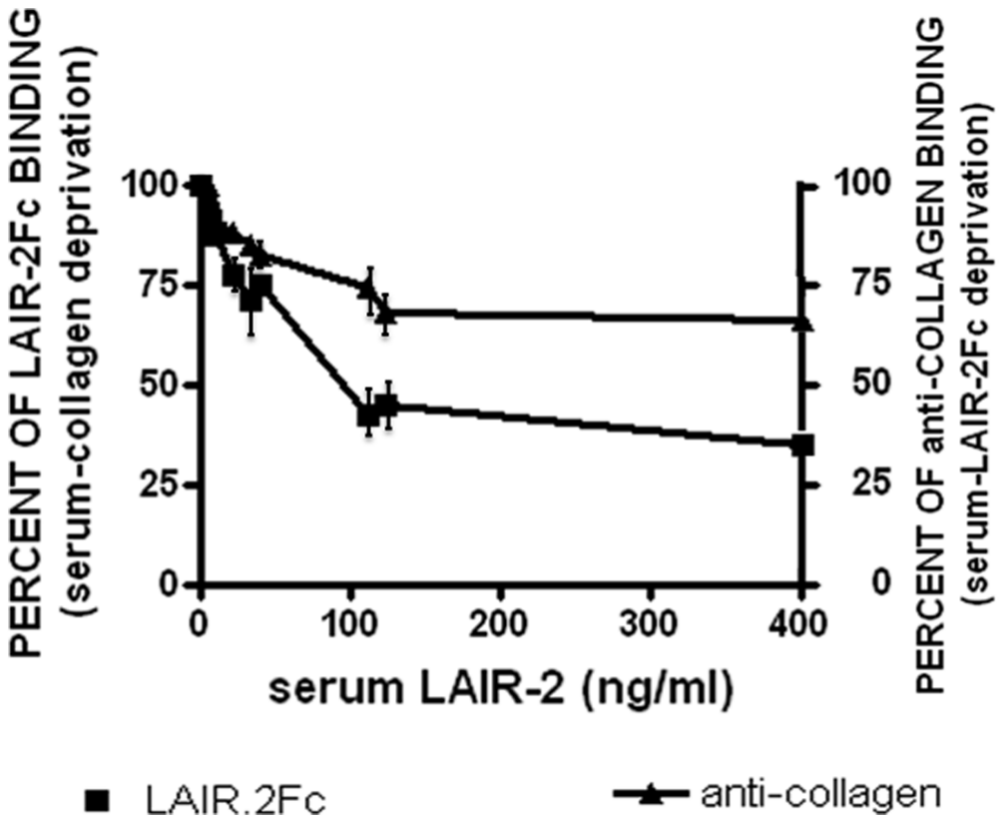
LAIR-2 found in serum of patients with ATD and healthy donors is able to recognize collagen as natural ligand. Pre-incubation of collagen solution with selected sera (4 HT; 4 GD; 4 MNTG) results in a decreasing of its ability to bind LAIR-2-Fc construct proportional to the sera LAIR-2 amounts. Similarly, pre-incubation of LAIR-2-Fc with sera shows a reduction of anti-collagen binding. Control wells were set up with the native (not pre-incubated) collagen solution or LAIR-2-Fc molecule (100% of binding). A representative experiment of three is shown. Bars represent standard deviation.

Finally, the specificity of the assay and the exclusion of a possible cross-reaction with soluble LAIR-1 (LAIR-1 Fc) were confirmed by dedicated experiments (Fig. 1A) and can be deduced by means of cross-inhibition experiments, which gave negative results, as depicted in Fig. 2.

### 3.2 LAIR-2 is Able to Bind Collagen *in vitro*

As shown in Fig. 2, pre-incubation of collagen solution in wells containing high LAIR-2 amounts results in marked decrease of its ability to bind LAIR-2-Fc. This does not occur following pre-incubation with sera containing low LAIR-2 amounts. Serum LAIR-2 concentration significantly correlated with collagen binding (p<0.0001, Spearman test). Thus, sera containing high LAIR-2 levels by ELISA have enhanced ability to bind the physiological ligand collagen.

In addition, pre-incubation of LAIR-2-Fc in wells containing sera with different amounts of LAIR-2 (measured by ELISA) results in a decrease of its ability to bind monoclonal antibodies specific to collagen (Fig. 2). Finally, in all cases, there is a residual capability of sera to bind, at least in vitro, collagen (Fig. 2). This can be due to serum LAIR-1 presence [6], [13].

## Discussion

Correct and appropriate functioning of the immune response depends on the finelly-regulated balance between activating and inhibitory signals. The inhibitory immune receptor family LAIR is expressed on most cells of the immune system, and its ligand collagen is ubiquitously expressed in the tissues. Therefore, triggering of the inhibitory immune receptor LAIR must be carefully regulated.

The identification of collagens as ligands for LAIR-1 revealed a novel function for extracellular matrix components as potential immune regulatory proteins [7], [9]. Subsequently, it was observed that this interaction could be blocked by LAIR-2, a secreted member of the LAIR-1 family [6]. An interesting implication of the discovery of LAIR-1 as an inhibitory collagen receptor is that tumor cells, known to upregulate collagen expression, may use this interaction to downregulate anti-tumor responses. Both extracellular matrix collagens and transmembrane collagens have been reported to be produced by tumor cells and/or tumor stroma. In addition, LAIR-2 molecules bind the same collagen molecules as the transmembrane inhibitory receptor LAIR-1 and this soluble receptor can block the interaction of LAIR-1 to both transmembrane and extracellular collagens. Hence, LAIR-2 may function as a competitor for human LAIR-1 in vivo, thereby regulating the inhibitory potential of this receptor.

Interestingly, LAIR-2 levels were demonstrated to be elevated in the joints of patients suffering from rheumatoid arthritis (RA) when compared to patients diagnosed with osteoarthritis [6], [11]. RA is a chronic autoimmune disease characterized by a persistent inflammation of the joints, which results in chronic tissue destruction. On the contrast, osteoarthritis is a result of both mechanical and biologic events that destabilize the normal coupling of degradation and synthesis of articular cartilage and are frequently characterized by a moderate inflammation. Thus, LAIR-2 may function as a pro-inflammatory mediator by decreasing the inhibitory potential of the immune inhibitor LAIR-1, resulting in enhanced activation of immune cells, a characteristic of autoimmune diseases.

ATD are prototypic organ-specific autoimmune diseases. Autoimmune thyroiditis is, characterized by marked mononucleate cell infiltration of the thyroid gland, with thyroid cell destruction and thyroid structure remodelling, eventually resulting in hypothyroidism. Graves’ disease causes hyperthyroidism mediated by thyroid-stimulating TSH-receptor antibodies, moreover extrathyroid tissue involvement (notably orbital structures in Graves’ ophthalmopathy) is often present. The collagen-rich extracellular matrix is important for maintenance of tissue structure, cell adhesion and migration during growth, differentiation, morphogenesis and lesion healing [15]. Collagen is a fibroblast cell membrane protein and serum antibodies against collagen (at least against the collagen XIII) have been linked to recent onset, congestive ophthalmopathy in patients with Graves’ ophthalmopathy [16].

Given the broad LAIR-1 expression on immunocytes [5]–[10] and the abundance of its ligand, collagen, and the capability of LAIR-2 to compete to LAIR-1, the interaction of LAIR-2 with collagen is probably relevant for immunoregulation in both normal and pathological conditions. We show that LAIR-2 serum levels are at least 100 fold higher in ATD subjects than in controls. Increased LAIR-2 levels in ATD could be associated with and contribute to chronic inflammation characterizing organ-specific autoimmunity. That this effect is due to altered thyroid function *per se* is unlikely, given that non-autoimmune hyperthyroid subjects (MNTG) studied for control showed LAIR-2 levels comparable to healthy individuals. LAIR-2 may function by down-modulating the inhibitory potential of LAIR-1. This event may prevent the binding of collagen to LAIR-1 thus resulting in immune activation. In support of this hypohesis, endogenous LAIR-2 has been shown to bind the extracellular matrix of chorionic villi *ex vivo* [17].

Of note, both soluble LAIR-1 (sLAIR-1) and LAIR-2 have been detected in the synovial fluid and urine of RA patients [11]. In addition, detectable levels of sLAIR-1 in the serum of healthy control subjects and increased levels in the sera of patients with hemorrhagic fever and renal syndrome [14]. This suggests a possible role of both of sLAIR-1 shedding and increased LAIR-2 production in the regulation of LAIR-1–induced inhibition, at least in these cases. Data on sLAIR-1 concentrations in sera from AITD patients are at present not available to us. However, monomer LAIR-2 was found to bind collagen with a higher affinity than does the membrane form of LAIR-1 [11], and to have a higher affinity for collagens than does sLAIR-1 [18].

Thus, LAIR-2 is likely to play a pivotal regulatory role of LAIR-1 function on T cells This might be a quite general mechanism, and the role of LAIR-2 in diseases with chronic inflammation, including allergic diseases, is worth of investigations.
